# The effect of muscle weakness on the capability gap during gross motor function: a simulation study supporting design criteria for exoskeletons of the lower limb

**DOI:** 10.1186/1475-925X-13-111

**Published:** 2014-08-04

**Authors:** Maarten Afschrift, Friedl De Groote, Joris De Schutter, Ilse Jonkers

**Affiliations:** 1 Human Movement Biomechanics Research Group, Department of Kinesiology, KU, Leuven, Belgium; 2 Production Engineering, Machine Design and Automation, Department of mechanical engineering, KU, Leuven, Belgium

**Keywords:** Assistance as needed, Muscle weakness, Design criteria, Exoskeleton, Musculoskeletal model, Simulation

## Abstract

**Background:**

Enabling persons with functional weaknesses to perform activities of daily living (ADL) is one of the main challenges for the aging society. Powered orthoses, or exoskeletons, have the potential to support ADL while promoting active participation of the user. For this purpose, assistive devices should be designed and controlled to deliver assistance as needed (AAN). This means that the level of assistance should bridge the capability gap, i.e. the gap between the capabilities of the subjects and the task requirements. However, currently the actuators of exoskeletons are mainly designed using inverse dynamics (ID) based calculations of joint moments. The goal of the present study is to calculate the capability gap for the lower limb during ADL when muscle weakness is present, which is needed for appropriate selection of actuators to be integrated in exoskeletons.

**Methods:**

A musculoskeletal model (MM) is used to calculate the joint kinematics, joint kinetics and muscle forces of eight healthy subjects during ADL (gait, sit-to-stand, stand-to-sit, stair ascent, stair descent). Muscle weakness was imposed to the MM by a stepwise decrease in maximal isometric force imposed to all muscles. Muscle forces were calculated using static optimization. In order to compensate for muscle weakness, ideal moment actuators that represent the motors of an exoskeleton in the simulation were added to deliver AAN required to perform the task.

**Results:**

The ID approach overestimates the required assistance since it relies solely on the demands of the task, whereas the AAN approach incorporates the capabilities of the subject. Furthermore, the ID approach delivers continuous support whereas the AAN approach targets the period where a capability gap occurs. The level of muscle weakness for which the external demands imposed by ADL can no longer be met by active muscle force production, is respectively 40%, 70%, 80% and 30%.

**Conclusions:**

The present workflow allows estimating the AAN during ADL for different levels of muscle weakness, which can be used in the mechatronic design and control of powered exoskeletons. The AAN approach is a more physiological approach than the ID approach, since the MM accounts for the subject-specific capabilities of the user.

## Background

Enabling persons with functional weaknesses to perform activities of daily living (ADL) is one of the main challenges for the aging society. It will be crucial for the welfare of our society to maintain the mobility and activity of the population as long as possible. Powered orthoses, or exoskeletons, have the potential to support ADL while promoting active participation of the user. This is essential to maintain neuromotor function and prevent disuse [1]. For this purpose, assistive devices should be designed and controlled to deliver the level of assistance needed for a specific individual during a specific motor activity. This level of assistance should bridge the capability gap, i.e. the discrepancy between the motor capabilities of the subject and the task requirements [2]. The motor capabilities are the joint moments that the subject is able to generate through coordinated muscle action. The task requirements are the external moments and powers that are imposed to the joints during the task execution. Hence, quantification of the capability gap is needed for appropriate selection of actuators to be integrated in lower-limb exoskeletons.

Currently, the actuator selection for assistive devices is mainly based on inverse dynamics (ID) calculations of joint moments and joint powers [3-5]. This method only accounts for the task requirements and assumes continuous support, despite the actual level of assistance required by the patient. Therefore, this method leads to overdimensioning of the actuators if the users have some remaining muscle function. As a first step towards defining assistance as needed (AAN), Carmichael et al. introduced the concept of a task model [6] that determines the task requirements and a strength model [7] that determines the motor capabilities of a person in reaching and lifting tasks. Using both these models, the required assistance for the upper limb can be calculated. However, assistance to support motor functions of the lower limb during ADL has not yet been evaluated using these concepts. Furthermore, the study of Carmichael et al. only considers assistance of the end effector (i.e. the hand). However, most lower limb exoskeletons are designed to support the function of the individual joints. Hence, the capability gap needs to be defined at the joint level in different ADL.

The purpose of the present study is to identify the capability gap during ADL. More specifically, we will investigate to what extent the task requirements can no longer be met if different levels of muscle weakness are present. To do so, the force generating capacities of the muscles in a musculoskeletal model (MM) will be modified to reflect different levels of weakness. Inherently, these models account for bi-articular muscle action and their inter-joint coupling as well as for the influence of the kinematic state on the force-generating capacities of the muscles. This approach allows quantifying the capability gap depending on the specific task requirements imposed by the ADL. The capability gap can then be expressed in terms of the joint moment deficits, for use in the mechatronic design of assistive devices. Furthermore, based on the task requirements and the capability gap for different levels of muscle weakness, critical tasks (i.e. the tasks that impose the highest demands) for a specific degree of freedom (DOF) can be identified and identify the critical threshold of muscle weakness that will interfere with ADL task execution.

In general, we hypothesize that the working envelope for the AAN design requirements in the presence of muscular weakness will differ substantially from the design requirements defined using the classic ID approach.

## Methods

### Experimental data

**Figure 1:**
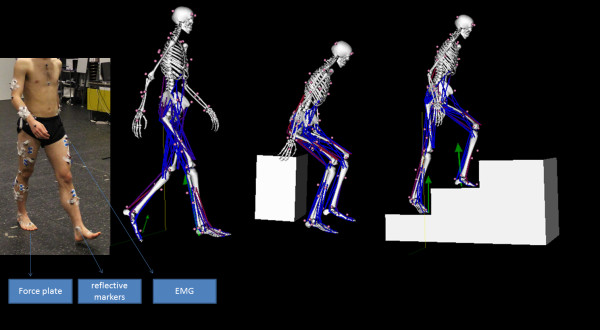
**Experimental set-up.** The left picture shows a subject during the experiments. Reflective markers were mounted on anatomical location of the subject to capture the motion with 10 infrared cameras. Muscle activity of 14 muscles is measured with surface electromyography. Ground reaction forces between the feet and the ground are measured with three force plates. These measurements are used with a generic musculoskeletal model in opensim to calculate the joint kinematics and kinetics during sit-to-stand, stand-to-sit, gait, stair ascent and stair descent.

Eight healthy subjects (61 +/− 8 kg, 23 +/− 3 years) provided written informed consent in accordance with the ethical committee of UZ Leuven to participate in this study. Each subject performed the selected ADL three times (Figure 1). The selected ADL are: sit-to-stand, stand-to-sit, gait, stair ascent, stair descent. The sit-to-stand movement is measured at self-selected speed in four conditions: a chair height of 100% knee height and 120% knee height, with and without hand support. A crutch of 75 cm height is used in the hand support condition. The gait data is measured overground at self-selected speed and on a split-belt treadmill at 3 and 5 km/h. The stair ascent and descent is measured using a staircase with 20 cm height and 40 cm width for each step. Motion data is collected with 10 Vicon cameras, sampled at 100 Hz. Ground reaction forces are measured using three force plates (Amti), sampled at 1000 Hz. The muscle activity of the rectus femoris, vastus lateralis, biceps femoris medialis, tibialis anterior, soleus and gastrocnemius lateralis are measured with surface electromyography (EMG, zero wire, Aurion, It), sampled at 1000 Hz. The SENIAM EMG placement protocol was used [8]. The EMG data were band-pass filtered (20–400 Hz), rectified and low pass filtered with a cutoff frequency of 10 Hz.

### Modeling and simulation

Opensim 3.1 is used for the dynamic simulations of human movement [9]. A generic musculoskeletal model (MM) with 23 degrees of freedom (DOF) and 92 musculotendon actuators was scaled to match the anthropometry and weight of the subject (name MM: 3DGaitModel2392) [10]. The scaled model reproduced the measured motion of the markers by means of an inverse kinematics algorithm that minimizes the squared distances between the measured markers and the markers on the scaled model. The motion of the model and the external forces from the force plates were used to calculate the joint moments with inverse dynamics (Opensim 3.1). The joint moments were normalized to the body mass of the subject. Muscle forces were calculated using the dedicated static optimization in Opensim. The goal function of the optimization solves the muscle redundancy problem by minimizing the sum of muscle activation squared.

### Simulation of muscle weakness

The simulations of muscle weakness were performed by a stepwise decrease of the maximal isometric force of the muscles in the MM ranging from 0 to 100% in steps of 10%. Decreasing the maximal isometric force of a muscle scales the force-length and force-velocity curves of the Hill-type muscle model. We assume an identical reduction in maximal isometric forces for all the muscles [11]. To compensate for muscle weakness, ideal moment actuators were added to the joints, which represent the motors of an exoskeleton in the simulation that would be available to deliver the assistance that is needed to successfully perform the task. The cost function of the static optimization, that minimizes the summed squared muscle activations, was supplemented with an additional term to account for the ideal moment actuators:

Constraint function:

$$\tau_{k}=\sum_{t=1}^{\;\mathrm{nMA}}\tau_{k,t}^{0}\;a_{t}+\;\sum_{m=1}^{N}\left[a_{m}\;f\left(F_{m}^{0},l_{m},v_{m}\right)\right]\;r_{m,k}$$

Objective function:

$$J\;=\;\sum_{t=1}^{\mathrm{nMA}}{\left(a_{t}\right)}^{2}+\sum_{m=1}^{N}{\left(a_{m}\right)}^{2}$$

nMa: Number of ideal moment actuators

k: The selected specific DOF of the MM

$$\tau_{t,k}^{0}$$

*: Optimal moment of the ideal moment actuator on the DOF k*

a_t_: Activation of the ideal moment actuator

N: Number of muscles in the MM

a_m_: Activation of the muscle

*F*^*0*^_*mv*_*: Maximal isometric force of the muscle*

l_m_*: Length of the muscle*

*V*_*m*_*: Velocity of the muscle fiber*

r_m,k_*: Moment arm of the muscle on the DOF k*

We assigned an optimal moment of $\tau_{t,k}^{0}$ = 1 Nm to the ideal moment actuators, therefore making them costly to use (i.e. high activations are required to use this actuator). Hence, actuation by muscles is preferred over actuation by the ideal moment actuators.

### Representation of the results

The analysis of the simulation results mainly focused on the sagittal plane joint moments, since for the tasks studied, these moments clearly exceed the joint moments required in other movement planes. For each subject the task requirements and capability gaps were calculated for the different ADL. The task requirements, i.e. the joint moments and joint powers imposed by the task to the joints, were calculated with inverse dynamics in Opensim. The maximal task requirement is identified as the maximal joint moment observed during the spectrum of ADL analyzed in this study. The capability gap is calculated by subtracting the joint moment generated by the muscle actuators in the MM (motor capability) from the joint moment imposed by the task (task requirements) and corresponds to the joint moment generated by the ideal moment generators. The capability gap for the different ADL was calculated for the different levels of muscle weakness. The maximal capability gap reflects the maximal joint moment deficit observed during the spectrum of ADL analyzed in this study. The maximal capability gap was calculated for each level of muscle weakness.

### Statistical analysis

The task requirements and capability gaps were compared for the different ADL and DOF using repeated measures Anova (significance threshold 5%). A Tukey's hsd post hoc test is used for pairwise comparison (significance threshold 5%). A two-sided Wilcoxon sign rank test with a significance threshold 5% was used to statistically test the differences in joint moments due to speed during gait and due to chair height and hand support during sit-to-stand. Comparing the measured EMG with calculated muscle activity is a method to validate the simulations [12]. Therefore, to check the validity of the simulation, the time series of the simulated muscle activations and the muscle activity, measured with EMG, were compared using a spearman correlation (Table 1).

**Table 1:** Correlation between measured muscle activity and simulated muscle activation

	**Gait**	**Stair ascent**	**Stair descent**	**Sit-to-stand**	**Stand-to-sit**
Rectus femoris	−0.09	0.44**	0.40**	−0.35	−0.35
Vastus lateralis	0.28**	0.83**	0.30**	0.89**	0.89**
Biceps femoris medialis	0.82**	0.49**	0.36**	0.93**	0.46**
Tibialis anterior	0.83**	0.66**	0.09	0.61**	0.35**
Soleus	0.66**	0.63**	0.22*	0.97**	0.73**
Gastrocnemius	0.76**	0.87**	0.25**	0.19*	0.15

*p < 0.05 **p < 0.01.

This table presents the spearman correlation coefficients for the correlation between the time series of the measured muscle activity and simulated muscle activation.

## Results

### Task requirements during ADL

**Figure 2:**
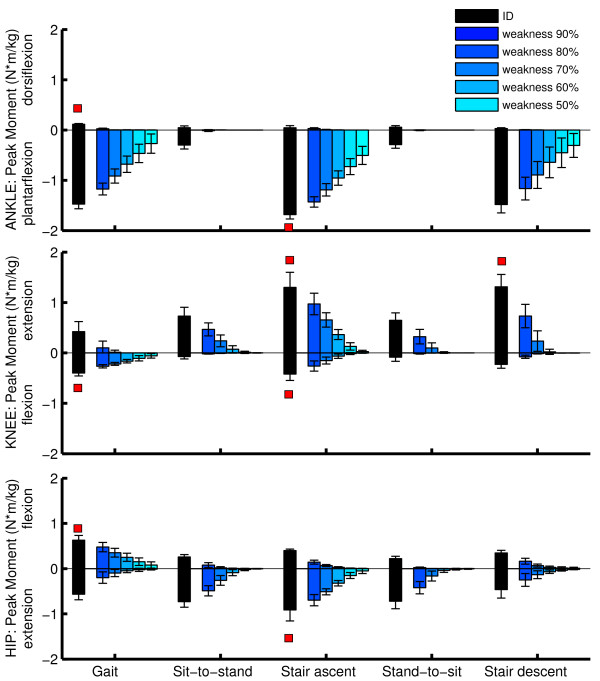
**Task requirements and capability gaps during ADL.** The task requirements for the ADL are shown in black (task requirements). The capability gaps for the different levels of muscle weakness are shown in blue. Gait is measured at self-selected speed and the sit-to-stand and stand-to-sit movements are measured with a chair of 100% knee height. The peak ID moment for the DOF were compared between the different movements using one way repeated measure Anova. A Tukey's hsd test is used for pairwise comparison when the one way repeated measures Anova test was significant significance threshold 5%. For the selected DOFs, the movement with the highest task requirements is marked with a red square (p < 0.05).

**Figure 3:**
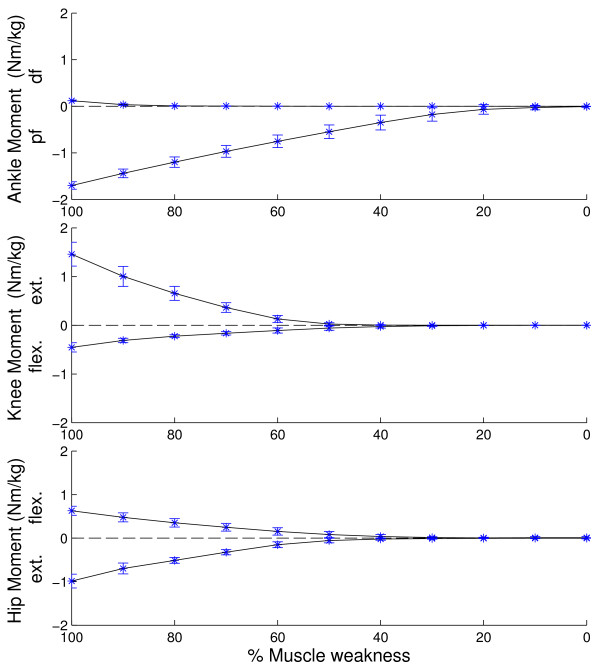
**Maximal task requirements and maximal capability gaps in terms of joint moments.** The maximal task requirements are the joint moments when 100% of muscle weakness is present. The maximal capability gap observed during the spectrum of daily living activities is shown as a function of the level of muscle weakness.

**Figure 4:**
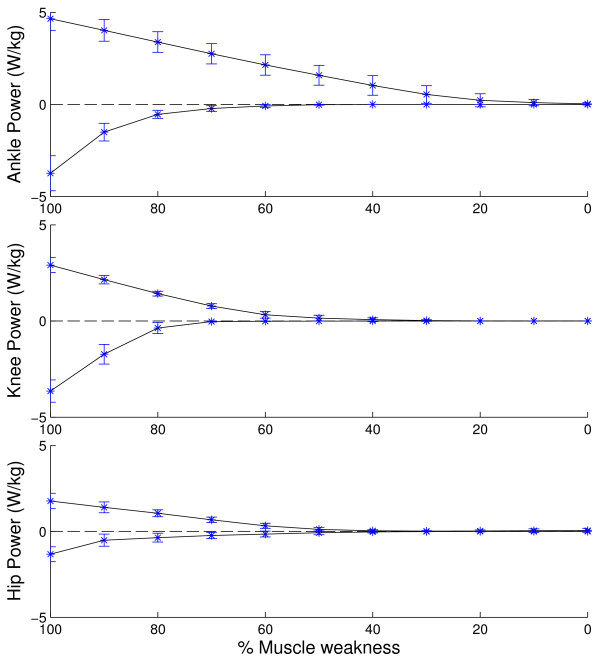
**Maximal task requirements and maximal capability gaps in terms of joint powers.** The maximal task requirements are the joint powers when 100% of muscle weakness is present. The maximal capability gap observed during the spectrum of daily living activities is shown as a function of the level of muscle weakness.

The task requirements differ between ADL and joints (Figure 2). Based on the task requirements we can specify the maximal task requirements in terms of joint moments (Figure 3) and joint powers (Figure 4). At the ankle joint, the stair ascent task imposes the highest net plantarflexion moment (1.68 +/− 0.09 Nm/kg, p < 0.05) whereas gait imposes the highest dorsiflexion moment (0.11 +/− 0.02 Nm/kg, p < 0.05). At the knee joint, the stair ascent and descent tasks impose the highest extensor moment (1.30 +/− 0.30 Nm/kg, 1.31 +/− 0.25 Nm/kg, p < 0.05) whereas the stair ascent and gait tasks impose the highest flexor moment (0.40 +/−0.06 Nm/kg, 0.42 +/− 0.13 Nm/kg, p < 0.05). At the hip joint, the stair ascent task imposes the highest extensor moment (0.91 +/− 0.25 Nm/kg, p < 0.05) whereas gait imposes the highest flexor moment (0.63 +/− 0.10 Nm/kg, p < 0.05). In general, considering the selected ADL, the ankle plantarflexion moments are highest (p < 0.01).The maximal task requirements in terms of joint power are shown in Figure 4. The highest positive ankle power is present during stair ascent (4.66 +/− 0.63 W/kg) and the highest negative power is present during stair descent (3.74 +/− 0.95 W/kg). For the knee joint, stair ascent imposes the highest positive power (2.91 +/− 0.39 W/kg), whereas stair descent imposes the highest negative power (3.64 +/− 0.58 W/kg). For the hip joint, stair ascent (1.73 +/− 0.49 W/kg) and gait (1.33 +/− 0.42 W/kg) impose the highest positive power, whereas stand-to-sit imposes the highest negative power (1.26 +/− 0.41 W/kg). In general, considering the ADL, the ankle joint positive power and the ankle joint and knee joint negative power are highest.

### Capability gap

**Figure 5:**
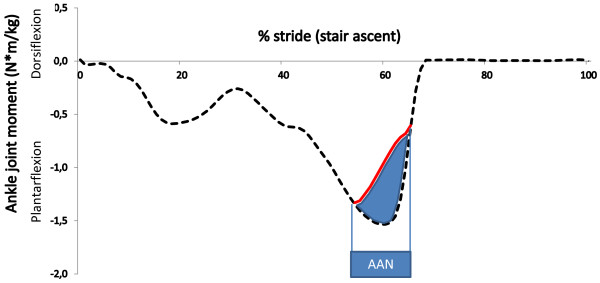
**Plantarflexion capability gap during stair ascent.** The difference between the net moment imposed by the task to the joint (task requirements, black dashed line) and the joint moment generated by the muscles (capability, red solid line) equals the capability gap (blue area). The capability gap is the external assistance that is needed to perform the task. In this case, the capability is calculated with a MM presenting 70% muscle weakness.

**Figure 6:**
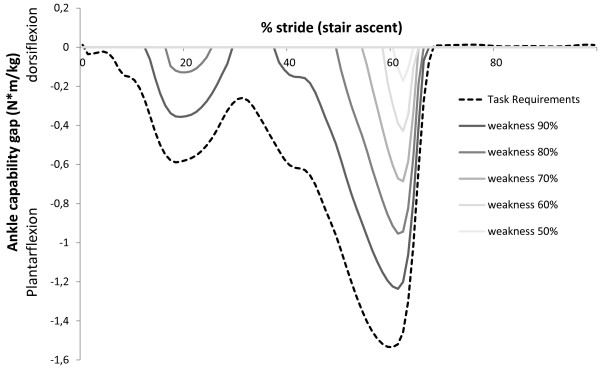
**Influence of muscle weakness on the plantarflexion capability gap during stair ascent.** The net ankle joint moment (task requirements, black dotted line) and the capability gap for different levels of muscle weakness (grey solid lines) are shown during a stair ascent stride. Both the level and timing of the capability gap increases when the level of weakness increases.

Figure 5 illustrates the main differences between the ID and AAN approaches during a stair ascent task for 70% muscle weakness. Firstly, the ID approach requires a higher assistive ankle moment (1.53 Nm/kg) compared with the capability gap in the AAN approach (0.68 Nm/kg). Secondly, the ID approach delivers continuous support whereas the AAN approach targets the period where a capability gap occurs (i.e. 55%-65% of the stair ascent cycle). Figure 6 illustrates the influence of an increasing level of muscle weakness on the capability gap during stair ascent. Both the timing and the magnitude of the capability gap depend on the level of muscle weakness.Figure 2 illustrates the capability gap for different ADL and different levels of muscle weakness. For example in the presence of 70% muscle weakness, no ankle dorsiflexion moment deficit is found. The highest plantarflexion capability gap occurs during stair ascent (0.96 +/− 0.14 Nm/kg, p < 0.05). For the knee joint, the highest knee extensor capability gap occurs during stair ascent (0.36 +/− 0.09 Nm/kg, p < 0.05) and the highest knee flexion capability gap occurs during gait (0.16 +/− 0.03 Nm/kg, p < 0.05). For the hip joint, the highest extensor capability gap occurs during stair ascent (0.32 +/− 0.06 Nm/kg, p < 0.05) and the highest flexion capability gap during gait (0.25 +/− 0.09 Nm/kg, p < 0.05).

A capability gap is found from 30% of muscle weakness onwards: from this level the ankle plantarflexion function during stair ascent and stair descent becomes insufficient and ideal moment generators assist. During gait, a capability gap is found when 40% of muscle weakness is present, and plantarflexion function is lacking. The sit-to-stand and stand-to-sit movements are less affected by overall muscle weakness: the knee and hip extension functions are only impaired when 70% of muscle weakness is present for the sit-to-stand task and when 80% of muscle weakness is present for the stand-to-sit task.

**Figure 7:**
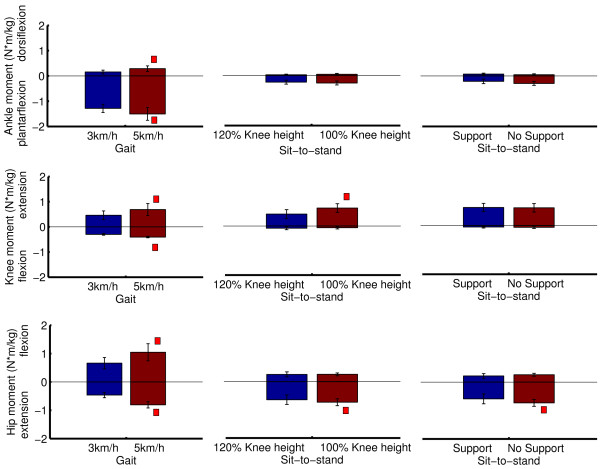
**The influence of more strenuous activities on the task requirements.** Increasing the gait speed (3 km/h vs. 5 km/h) causes an increase in the task requirements. There is an increase in net plantarflexion moment (p < 0.01), dorsiflexion moment (p < 0.05), knee extension moment (p < 0.05), knee flexion moment (p < 0.01), hip flexion moment (p < 0.01) and hip extension moment (p < 0.01). Increasing the chair height from 100% knee height to 120% knee height decreases the net knee extension and hip extension moment (p < 0.05, p < 0.05). The use of hand support causes a decrease in peak hip extension moment (p < 0.05). The peak joint moments were compared using a Wilcoxon sign rank test. The significant differences between the conditions are marked with a red square.

The capability gap differs significantly depending on the specific DOF: the ankle plantarflexion function is impaired from 30% weakness onwards, whereas the dorsiflexion function is only impaired when 90% weakness is present. For the knee and hip joint, the knee flexion/extension and hip flexion/extension functions are impaired during ADL from 50% muscle weakness onwards.Based on the capability gaps for the ADL, we can specify the maximal capability gap for the different DOF in term of joint moments (Figure 3) and joint powers (Figure 4). These figures show the maximal moment and power assistance that is needed to perform the ADL for the different levels of muscle weakness.When inducing more strenuous activities, the task requirements for several DOF increase (Figure 7). For example increasing the walking speed from 3 km/h to 5 km/h causes an increase in the task requirements for all the DOF.

## Discussion

The aim of the present study was to identify the capability gap during ADL when muscle weakness is present. We hypothesized that the AAN paradigm based on an MM differs substantially from the classic ID approach. The findings of the study can be used to define the specifications of the actuators to be integrated in powered assistive devices. Similar to the study of Carmichael et al. [2], we have used an MM to confront the force generating capacities of subjects when different levels of muscle weakness were introduced. This study explores the capability gap of the lower limbs during gross motor function related to typical ADL. Based on the capability gap; the level of assistive moments for each DOF can then be specified. The maximal capability gap in terms of moment or power can be used to select the actuators of an assistive device. Furthermore, the joint moment capability gap for a given ADL can be used to provide assistance as needed during the task execution through appropriate control of the assistive device.

### Comparison between the ID approach and the AAN approach

**Figure 8:**
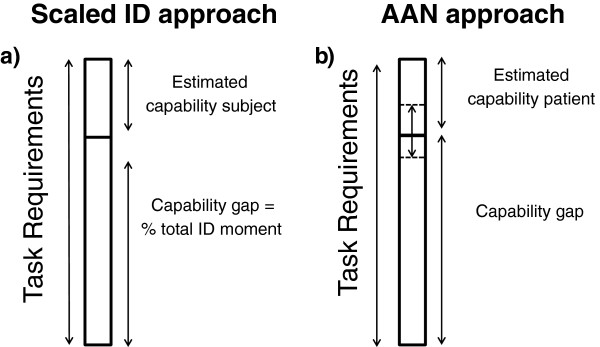
**Scaled ID approach versus AAN approach. (a)** The scaled ID approach uses an estimation of the capability of the subject, which is the constant scaling factor of the peak task requirement. **(b)** The AAN approach estimates the capability of the subject using an MM. Due to the musculotendon properties and the coupling between joints in the MM, the relative capability with regards to the ID moment varies with the kinematics and the task requirements at the other joints. Therefore the capability varies in time during ADL.

Our findings confirm a substantial capability gap that depends on the specific ADL task and the level of muscle weakness. The capability gap is determined based on a joint moment deficit, illustrated in Figure 5. This figure emphasizes the difference between the ID approach and the AAN approach. The ID approach overestimates the required assistance as it relies solely on the demands of the task, whereas the AAN approach incorporates the capabilities of the subject. To date, several exoskeletons are designed based on a percentage of the peak joint moments that are calculated with the ID approach [13]. This method assumes that the capability of the subject is a constant percentage of the peak moment calculated with ID (Figure 8a). However, not only the task requirements, but also the angle dependency of the moment generating capacity around the joint and the task requirements of the adjacent joints will affect the capability gap. The use of MM–based dynamic simulations accounts for the force-length properties of the muscle fibers and for the change in moment arm of the muscle with the joint angle. Therefore, the moment generating capacity of the subject is not constant, but varies in magnitude during the motor task in the AAN approach and affects the capability gap (Figure 5 + Figure 8b). This causes a substantial difference between required assistance in the AAN approach and the (scaled) ID approach.

### Capability gap during ADL

The simulation of muscle weakness allows us to identify to what extent the MM is able to cope with muscle weakness. The capability gaps are analyzed for the ankle and knee in the sagittal plane and for the hip in the sagittal, frontal and transversal plan. The MM is able to perform stair ascent until 30% of muscle weakness is present, which is caused by a plantarflexion capability gap. The tolerance of the MM-model to muscle weakness, i.e. level of muscle weakness for which the external demands imposed by gait, sit-to-stand, stand-to-sit and stair descent can no longer be met by active muscle force production, is respectively 40%, 70%, 80% and 30%. The tolerance level reported for gait agrees with previously reported simulation results [14]. The tolerance level for the sit-to-stand movement is comparable to the experimental results from Hughes et al. [15]. In the presence of a reduction of 63% in maximal isometric force, elderly had to deliver 97% of their available strength to rise from the chair. Despite this agreement with experimental work, the tolerance level of 70% of muscle weakness for the sit-to-stand movement is somewhat surprising as difficulties in executing sit-to stance maneuvers are frequent in the elderly population [15]. These results indicate that in the elderly population, instability and inadequate coordination strategies more than muscle strength impair task execution when rising from a chair, as previously suggested by [16].Several factors influence the assistance that is needed to perform ADL. Firstly, the level of muscle weakness determines the timing and the magnitude of the capability gap (Figure 6). Secondly, the magnitude of the capability gap varies between the tasks and the joints. The plantarflexion has the highest maximal capability gap for all the DOF. This is caused by the high plantarflexion moments that are imposed during stair ascent (1.70 Nm/kg). These results emphasize the importance of plantarflexion assistance in powered exoskeletons, in particular during stair ascent when muscle weakness is present. Furthermore, the high angular velocity during push off in combination with the high plantarflexion joint moments causes high positive joint powers. Therefore, generating sufficient plantarflexion moment and power will be an important challenge in the design of lower limb exoskeletons.The crucial activity for the dorsiflexion capability gap is gait. Despite the relative small dorsiflexion moment deficits during gait, assisting this DOF is important for patients with drop foot gait. Our results show that there is only 0.11 (+/−0.02) Nm/kg dorsiflexion moment required to assist persons with total central paralysis of the dorsiflexion muscles during the selected ADL. However, the required dorsiflexion moment increases with gait speed (Figure 7).

In contrast with the ankle, the directional dominance of the assistance is less clear at the knee and hip joint: at the knee a slight dominance of knee extension assistance is confirmed whereas at the hip both flexion and extension require similar assistance. Furthermore, the level of weakness tolerated before introducing the moment generators is substantially higher at the knee and hip.

### Limitations

The aforementioned results should be interpreted and used within the limitations of the methodology. Firstly, to assess the validity of the simulations, we have calculated the correlation between the simulated muscle activations and the measured muscle activity (Table 1). These results indicate that the simulated muscle activation of all the mono-articular muscles correlates significantly with the measured activity. It is known that the activity of the bi-articular rectus femoris (RF) is less well predicted in the simulation [17]. Secondly, we assume in our simulation that persons perfectly adapt to an assistive moment, i.e. subjects are assumed not to oppose the induced external moment by increasing their antagonistic muscle activity or change the joint kinetics pattern. Indeed, experiments with single joint powered exoskeletons have shown that there is no difference in joint kinetics with and without assistance in healthy subjects [18,19]. Furthermore, there is no difference in antagonistic muscle activity but a decrease in agonistic muscle activity when walking with a powered exoskeleton [20,21]. Therefore, the assumption seems to be valid. Thirdly, the current workflow assumes that the MM represents the subject specific impairments of user/patient. It is known that aging and pathologies cause changes in musculotendon properties and activation dynamics [22]. In order to estimate the capability for these populations, the MM should ideally be adjusted towards the target population. Fourthly, the presented results rely on one specific objective function. However, the influence of the cost function on the capability gap is limited. The capability gap is the moment deficit when muscles maximally contribute to the desired motion. Therefore the influence of the cost function, which merely defines the muscle force distribution and not the overall level of agonistic muscle activity, on the moment deficit is small. Lastly, this study does not account for the stabilization of joints, fatigue, potential losses in body mass due to weakness and it assumes that the kinematics remain constant despite the presence of muscle weakness.

### Application of the results

Within the limitations of the study, the results can be used in the design of assistive devices that support ADL. Elderly and patients with sarcopenia have a reduction in maximal isometric force that ranges between 20%-40% [11]. Our results suggest that in these cases solely an assistive plantarflexion moment is required to perform ADL (Figure 3). However, this study does not include the influence of fatigue on the capability gap of the individual subject. An additional knee flexion/extension and hip flexion/extension moment is required when the level of muscle weakness increases to 50%. In the study of Stevens-Lapsley et al., there is a reduction of 50 +/− 20% in maximal isometric force in persons with severe Parkinson disease, with 68% of the patients with severe Parkinson disease have 70% or less reduction in maximal isometric knee extensor moment [23]. Assuming an upper limit of body mass that equals 92.9 kg [23], Figures 3 and 4 can then be used to determine the design requirements of an exoskeleton that is able to assist this population in ADL: The exoskeleton should be able to deliver peak moments of 89.1 Nm (254 W) at the ankle joint, 33.4 Nm (72.5 W) at the knee joint and 29.7 Nm (63.17 W) at the hip joint. Furthermore, the present simulation workflow can be used to select the actuators of an assistive device with specific design criteria. The relative cost of the ideal moment actuators at the hip, knee, and ankle will influence bi-articular muscle action and therefore the capability gap at the joints. Therefore, the relative costs can be optimized for specific design criteria. For example, one could increase the cost of using the ideal moment actuator at the ankle joint in order to minimize the actuator size at the ankle (at the cost of increasing the size at the knee and hip).

### Further directions

Although the added value of the current results is without question, future work should concentrate on forward simulations that allow changes in the motor strategy during the task. For example, changing the strategy to stand up from a chair influences the kinematics and peak joint moments [24]. Hughes et al. described a difference in chair rise strategy in the functionally impaired elderly in order to increase their stability [16]. On the one hand, these differences in motor strategies could influence the required assistance during ADL. On the other hand, exoskeletons are designed to increase the physical performance of the user, in order to achieve or exceed a healthy motor function.

The current study provides sufficient proof-of-concept to consider the design of targeted functional protocols that allow tuning the control settings for the exoskeleton to the functional limitations in terms of muscle weakness of individual subjects. This AAN control will promote active participation of the user which is essential to prevent disuse and promote neuromotor rehabilitation.

## Conclusion

The combination of experimental measurements with an MM enables the identification of the capability gap during ADL when muscle weakness is present. Both the magnitude and timing of the assistance in this AAN approach differs substantially from the classic ID approach. This AAN is a more physiological approach, since it accounts for the capabilities of the operator which are modeled in the MM. The results can be used, within the limitations of the methodology, in the mechatronic design and control of an assistive device.

## Abbreviations

AAN: Assistance as needed; ADL: Activities of daily living; DOF: Degree of freedom; EMG: Electromyography; ID: Inverse dynamics; IK: Inverse kinematics; MM: Musculoskeletal model.

## Competing interests

The authors declare that they have no competing interests.

## Authors’ contributions

MA performed the experimental data collection, contributed to model implementation, executed the simulations, analyzed data and interpreted the data. FDG implemented the simulation model, contributed to data analysis and interpretation. IJ contributed to the data analysis and interpretation. JDS, FDG and IJ are senior authors who participated in the design of research and helped to draft the manuscript. All authors read and approved the final manuscript.

## References

[B1] Kaelin-lang A Sawaki L Cohen LG Role of Voluntary Drive in Encoding an Elementary Motor Memory J Neurophysiol 2005 93 2 1099 1103 15456807 10.1152/jn.00143.2004

[B2] Carmichael MG Liu D Estimating Physical Assistance Need Using a Musculoskeletal Model IEEE Trans Biomed Eng 2013 60 1912 1919 23380850 10.1109/TBME.2013.2244889

[B3] Skelton J Wu S Design of a Powered Lower-Extremity Orthosis for Sit-to-Stand and Ambulation Assistance J Med Devices 2013 7 1 2

[B4] Tsukahara A Kawanishi R Hasegawa Y Sankai Y Sit-to-Stand and Stand-to-Sit Transfer Support for Complete Paraplegic Patients with Robot Suit HAL Adv Robot 2010 24 1615 1638

[B5] Bleex EE Zoss AB Kazerooni H Chu A Biomechanical Design of the Berkeley Lower Trans Mechatron 2006 11 128 138

[B6] Carmichael MG Liu D A task description model for robotic rehabilitation Conf Proc Annu Int Conf IEEE Eng Med Biol Soc IEEE Eng Med Biol Soc Conf 2012 2012 3086 3089 10.1109/EMBC.2012.6346616 23366577

[B7] Carmichael MG Liu D Towards using Musculoskeletal Models for Intelligent Control of Physically Assistive Robots Eng Med Biol Soc 2011 1 8162 8165 10.1109/IEMBS.2011.6092013 22256236

[B8] Hermens HJ Freriks B Disselhorst-Klug C Rau G Development of recommendations for SEMG sensors and sensor placement procedures J Electromyogr Kinesiol 2000 10 361 374 11018445 10.1016/s1050-6411(00)00027-4

[B9] Delp SL Anderson FC Arnold AS Loan P Habib A John CT Guendelman E Thelen DG OpenSim: open-source software to create and analyze dynamic simulations of movement IEEE Trans Biomed Eng 2007 54 1940 1950 18018689 10.1109/TBME.2007.901024

[B10] Hamner SR Seth A Delp SL Muscle contributions to propulsion and support during running J Biomech 2010 43 2709 2716 20691972 10.1016/j.jbiomech.2010.06.025 PMC2973845

[B11] Doherty TJ Invited review: Aging and sarcopenia J Appl Physiol Bethesda Md 1985 2003 95 1717 1727 10.1152/japplphysiol.00347.2003 12970377

[B12] Crowninshield RD Use of Optimization Techniques to Predict Muscle Forces J Biomech Eng 1978 100 88 92

[B13] Cenciarini M Dollar AM Biomechanical considerations in the design of lower limb exoskeletons IEEE Int Conf Rehabil Robot Proc 2011 2011 5975366 10.1109/ICORR.2011.5975366 22275570

[B14] Van der Krogt MM Delp SL Schwartz MH How robust is human gait to muscle weakness? Gait Posture 2012 36 113 119 22386624 10.1016/j.gaitpost.2012.01.017 PMC4890623

[B15] Hughes MA Myers BS Schenkman ML The role of strength in rising from a chair in the functionally impaired elderly J Biomech 1996 29 1509 1513 8945648

[B16] Hughes MA Schenkman ML Chair rise strategy in the functionally impaired elderly J Rehabil Res Dev 1996 33 409 412 8895136

[B17] Duysens J De Groote F Jonkers I The flexion synergy, mother of all synergies and father of new models of gait Front Comput Neurosci 2013 7 14 23494365 10.3389/fncom.2013.00014 PMC3595503

[B18] Kao P-C Lewis CL Ferris DP Invariant ankle moment patterns when walking with and without a robotic ankle exoskeleton J Biomech 2010 43 203 209 19878952 10.1016/j.jbiomech.2009.09.030 PMC2813403

[B19] Lewis CL Ferris DP Invariant hip moment pattern while walking with a robotic hip exoskeleton J Biomech 2011 44 789 793 21333995 10.1016/j.jbiomech.2011.01.030 PMC3075111

[B20] Galle S Malcolm P Derave W De CD Gait & Posture Adaptation to walking with an exoskeleton that assists ankle extension Gait Posture 2013 38 495 499 23465319 10.1016/j.gaitpost.2013.01.029

[B21] Sawicki G Mechanics and energetics of level walking with powered ankle exoskeletons J Exp Biol 2008 211 1402 1413 18424674 10.1242/jeb.009241

[B22] Thelen DG Adjustment of Muscle Mechanics Model Parameters to Simulate Dynamic Contractions in Older Adults J Biomech Eng 2003 125 70 12661198 10.1115/1.1531112

[B23] Stevens-Lapsley J Kluger BM Schenkman M Quadriceps muscle weakness, activation deficits, and fatigue with Parkinson disease Neurorehabil Neural Repair 2012 26 533 541 22140196 10.1177/1545968311425925 PMC12930528

[B24] Yoshioka S Nagano A Himeno R Fukashiro S Computation of the kinematics and the minimum peak joint moments of sit-to-stand movements Biomed Eng Online 2007 6 26 17608922 10.1186/1475-925X-6-26 PMC1929086

